# Alcohol Induces More Severe Fatty Liver Disease by Influencing Cholesterol Metabolism

**DOI:** 10.1155/2019/7095684

**Published:** 2019-02-12

**Authors:** Bo Li, Shan-Shan Lei, Jie Su, Xia-Miao Cai, Hao Xu, Xinglishang He, Ye-Hui Chen, Hai-Xia Lu, He Li, Liu-Qing Qian, Xiang Zheng, Gui-Yuan Lv, Su-Hong Chen

**Affiliations:** ^1^Zhejiang University of Technology, Hangzhou, Zhejiang 310014, China; ^2^Zhejiang Chinese Medical University, Hangzhou, Zhejiang 310053, China; ^3^Wenzhou Medical University, Wenzhou, Zhejiang 325035, China

## Abstract

*Objectives*. Fatty liver disease (FLD) is a major cause of morbidity and mortality worldwide. Dietary cholesterol and alcohol consumption are important risk factors for the progression of FLD, but whether and how alcohol induces more severe FLD with cholesterol ingestion remain unclear. Herein, we mainly used the Lieber-DeCarli diet to establish the FLD mouse model to investigate the synergistic effects of alcohol and cholesterol metabolism on liver damage. The indices of aspartate transaminase (AST), alanine transaminase (ALT), low-density lipoprotein cholesterol (LDL-c), and total cholesterol (TC) levels, inflammation foci, and pathogenesis by hematoxylin and eosin (H&E) and Oil Red O staining revealed that alcohol induces more severe liver damage by influencing cholesterol metabolism, which might be primarily related to the influence of cholesterol absorption, synthesis, and excretion on the liver or small intestine. Moreover, inhibition of absorption of intestinal cholesterol, but not of fat, sucrose, and alcohol, absorption into the body's metabolism by Ezetimibe, significantly improved FLD in rats fed with the high fat-cholesterol-sucrose and alcohol diet. These results showed that alcohol plays an important role in cholesterol metabolism in FLD.

## 1. Introduction

Fatty liver disease (FLD), including mainly alcoholic fatty liver disease (AFLD) and nonalcoholic fatty liver disease (NFALD), is a major cause of morbidity and mortality worldwide. Morbidity of NAFLD is 15% in China and 12.9~46% worldwide, while AFLD is 4.5% in China [1]. The spectrum of FLD encompasses steatosis, steatohepatitis, progressive fibrosis, and hepatocellular carcinoma, and it is influenced by numerous factors including alcohol abuse, high-fat diet, and a high-cholesterol diet. Meanwhile, binge or chronic alcohol consumption and high-fat-cholesterol diet are popular trends in residential table manners.

A diet containing high cholesterol may induce FLD. Reports by our group [2] and others [3] suggest that dietary cholesterol is a critical factor in the development of experimental steatohepatitis in animal models. Animal experiments also have revealed that inflammation and steatosis in a NAFLD model rat treated with Ezetimibe, inhibiting exogenous cholesterol absorption, showed notable improvements [4, 5]. Epidemiological studies have also reported that dietary cholesterol consumption is independently associated with the development of FLD [6]. High-cholesterol diets induce FLD by impairing hepatocyte mitochondrial functions and causing oxidative stress, increasing lipid synthesis and lipidosis, inducing hepatic steatosis, steatohepatitis, etc. [2, 3]. Research has revealed that a high-fat diet without cholesterol induces a slight elevation of liver enzymes, such as aspartate transaminase (AST) and alanine transaminase (ALT), and no inflammatory infiltrate, whereas the high-fat diet, with cholesterol or cholesterol and alcohol, can cause more serious liver injury [3, 7]. In addition, a high-fat diet with alcohol can induce more lipid droplets compared with that without alcohol [8]. And it has been found that the increase of intestinal cholesterol absorption and low-density lipoprotein (LDL) production rate by alcohol may be important mechanisms for exacerbation of hypercholesterolemia in the cholesterol-fed rabbit model [9].

**Figure 1:**
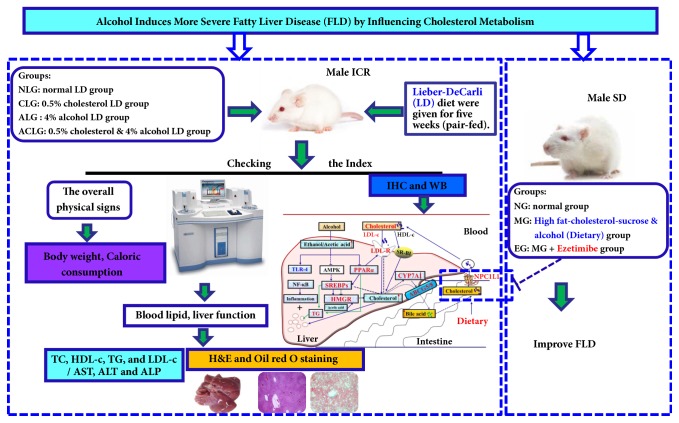
An overview of the experimental procedures.

These findings hinted that cholesterol and alcohol may have important synergistic effects on the development of FLD. And there were few researches involving the mechanism of how alcohol induces more severe FLD synchronously with cholesterol diet. Our aims were to determine whether and how alcohol together with high cholesterol interacts to induce more severe FLD. In this study, mice were fed a standard Lieber-DeCarli liquid diet (LD), LD plus 0.5% cholesterol diet, LD plus 4% alcohol diet, or LD plus 4% alcohol and 0.5% cholesterol diet to mimic diet habits in humans and to investigate the effect of alcohol consumption on cholesterol metabolism including its absorption, synthesis, and excretion. Additionally, we aimed to verify whether inhibition of intestinal cholesterol absorption, but not the absorption of fat, sucrose, and alcohol into the body's metabolism by Ezetimibe, can significantly improve FLD in rats fed with high fat-cholesterol-sucrose and alcohol or not. The experimental procedures are displayed in Figure 1.

## 2. Materials and Methods 

### 2.1. Materials and Reagents

Total cholesterol (TC), triglyceride (TG), high density lipoprotein cholesterol (HDL-c), low-density lipoprotein cholesterol (LDL-c), alanine transaminase (ALT), aspartate transaminase (AST), and phosphoric acid transaminase (ALP) kits were all purchased from Medical System Biotechnology Co., Ltd. (Ningbo, Zhejiang, China). Hematoxylin, Eosin, Masson, and Oil Red O were all purchased from Nanjing Jiangcheng Technology Co., Ltd. (JiangSu, China). Antibodies against toll-like receptor 4 (TLR-4), nuclear factor *κ*B p65 (NF-*κ*B p65), low-density lipoprotein receptor (LDLR), peroxisome proliferator-activated receptor alpha (PPAR*α*), sterol regulatory element-binding transcription factor 2 (SREBP2), sterol regulatory element-binding transcription factor 1 (SREBP1), cholesterol 7-alpha hydroxy-lase (CYP7A1), Niemann-Pick C1 Like 1 (NPC1L1), 3-hydroxy-3-methylglutaryl- Coenzyme A reductase (HMGCR), ATP-binding cassette G8 (ABCG8), ATP-binding cassette G5 (ABCG5), and GAPDH were all purchased from Santa Cruz Biotechnology, USA. Antibodies against scavenger receptor class B type I (SR-BI) were from Abcam (Cambridge, USA). HRP conjugated goat anti-rabbit IgG (PV-6001) and HRP conjugated goat anti-mouse/rabbit IgG (PV-6000) were from Zhongshan Goldenbridge Biotechnology Co. (Beijing, China).

### 2.2. Animals and Experimental Design

Forty-eight specific pathogen-free male ICR mice and thirty male SD rats were purchased from Zhejiang Academy of Medical Sciences (Hangzhou, China); the license number is SCXK (Zhe) 2014-0001. The housing facility is keeping with the national standards principles of GB14925-2010 (Laboratory Animal-Requirement of Environment and Housing Facilities) for laboratory. The care and experimental operation were conforming to the rules of “Zhejiang province Administration Rule of Laboratory Animal”. The mice were placed in three per cage in every group and pair-fed after one week of acclimation.

Mice were randomly divided into four groups (n=12) according to the weight and following as Normal LD group (NLG, feeding with the standard Lieber-DeCarli liquid diet), Cholesterol LD group (CLG, normal LD plus 0.5% cholesterol (*m/v*)), Alcohol LD group (ALG, 4% alcohol LD (*v/v*)), and Cholesterol and Alcohol LD group (CALG, 4% alcohol LD plus 0.5% cholesterol). Nutritional ingredient of the Lieber-DeCarli diet (LD) was prepared and mice were pair-fed as we described previously [10]. Caloric of these four LD diets was same (Table 1). The mice in different groups were fed with the corresponding diet for 5 weeks to set up FLD models. Throughout the experiment, body weight and caloric consumption were evaluated.

SD rats were firstly randomly divided into 3 groups (*n* = 10) according to the weight. The normal group (NG) received a standard pellet chow diet and water throughout for the first 4 weeks of the experiment. Meanwhile the high fat-cholesterol-sucrose and alcohol- (ACHFCSD-) induced rats (MG and EG) like as we described previously [11], given high fat-cholesterol-sucrose diet and 22% alcohol-water drinking for the first 4 weeks of the experiment, had a significant increase in ALT, AST, and TC (data not shown). For the next 12 weeks, MG rats were set as the model control group and continued to receive high fat-cholesterol-sucrose and alcohol; EG rats were given high fat-cholesterol-sucrose, alcohol, and Ezetimibe (at the doses of 1 mg/kg, p.o.). Throughout the experiment, body weight was evaluated (data not shown).

At the end of experiments, mice and rats were fasted overnight and blood was obtained from the ophthalmic venous plexus. The blood then was centrifuged at 3500 rpm/min for 10 min to get serum for biochemical analysis. At the end of experiment, the mice and rats were sacrificed via euthanasia and collected liver tissues. One part of livers and small intestines were put into 4% neutral buffered formalin and embedded in paraffin for hematoxylin-and-eosin (H&E), immunohistochemistry (IHC), or Masson's trichrome (Masson) staining. The remainder of the fresh livers were frozen in liquid nitrogen and stored at 80°C for Oil Red O staining and western blot analysis.

### 2.3. Determination of Serum Biomarkers

The serum lipid profile of TC, TG, LDL-c, and HDL-c and liver function biomarkers of ALT, AST, and ALP were measured with the corresponding kits by an automatic biochemical analyzer (TBA-40FR, Toshiba, Japan) as we described previously [11].

### 2.4. Hepatic Histopathological Evaluation by H&E, Oil Red O, and Masson Staining

Liver segments were fixed in 4% neutral buffered formalin solution for a minimum of 72 h and embedded in paraffin wax. Embedded liver tissues were cut at 4 *μ*m thickness sections and stained with H&E and Masson as we described previously [12, 13]. Took out a small part of liver tissues from −80°C and prepared the frozen OCT to embed liver. Embedded liver tissues were cut at 8 *μ*m thickness at −20°C and then stained with Oil Red O as we described previously [2, 10]. All staining was photographed with the biological microscope (BA410, Motic, China) and analysed with the Image-Pro Plus software to estimate lipid deposition, inflammation, and fibrosis lesion.

### 2.5. Western Blot Analysis

The western blot was similar as we described previously [14]. Briefly, 50~100 mg frozen liver tissues were mechanically lysed in liquid nitrogen and lysed in RAPI buffer (Solarbio, Beijing, China) with protease/phosphatase inhibitor (Cell Signaling Technology, Canada) for 30 min on ice. Then lysates were clarified by centrifugation at 12,000 rpm/min for 15 min at 4°C and the protein concentration was detected with a BCA protein assay kit (Beyotime, Jiangsu, China). After sample protein was mixed with 5 × Loading Buffer (Beyotime, Jiangsu, China) and degenerated at 95°C, equivalent amount of total protein of samples was separated by SDS-PAGE at room temperature and electrotransferred onto a polyvinylidene difluoride (PVDF) membranes in ice (Pall Corporation, Mexico). Membranes were blocked with 5% BSA for 2 h at room temperature and incubated overnight at 4°C with corresponding first antibody (solute in PBST) against the HMGCR, NF-*κ*B p65, or P NF-*κ*B p65 at dilutions of 1:1000. GAPDH (diluted 1:1000 in PBST) was used as a loading control. After washed three times for 5 min each in PBST, membranes were incubated with the appropriate secondary antibodies HRP conjugated goat anti-rabbit IgG (H+L) (Santa Cruz Biotechnology, USA) at dilutions of 1:1000 for 2 h at room temperature and washed again. The blotted protein bands were detected by chemiluminescent assay kit (Beyotime, Jiangsu, China) and the protein expression levels were normalized to GAPDH.

### 2.6. Immunohistochemistry (IHC) Analysis of Inflammation and Cholesterol Metabolism Nodes

The immunohistochemistry (IHC) staining was similar as we described previously [12]. The prepared paraffin liver or small intestine sections were coped with 3% H_2_O_2_ for preventing and inactivating endogenous catalase for 10 min at room temperature. Then sections had to go through antigen retrieval with citrate buffer liquid (Beyotime, Jiangsu, China). The samples were reacted with primary antibody of TLR-4, LDLR, SR-BI, NPC1L1, PPAR*α*, SREBP2, SREBP1, CYP7A1, ABCG8, and ABCG5 overnight at 4°C. Then the appropriate secondary antibodies (Zhongshan Goldenbridge Biotechnology Co., Beijing, China) were incubated for 30 min at room temperature. After DAB staining, the nucleus were stained by hematoxylin and sealed by neutral resins. Positive staining presented yellow or brown color under the biological microscope. The data of protein expressions were semiquantitative analysed as integrated option density (IOD) in positive area of the microphotograph with the Image-Pro Plus software.

### 2.7. Statistical Analysis

All values were expressed as the mean ± standard deviation. The data were subjected to one-way analysis of variance. Differences were considered statistically significant if the P value was less than 0.05. All analyses were performed using SPSS version 15.0 software.

## 3. Results

### 3.1. Alcohol with Cholesterol Diet Causes Less Weight Gain Than Cholesterol Diet

**Figure 2:**
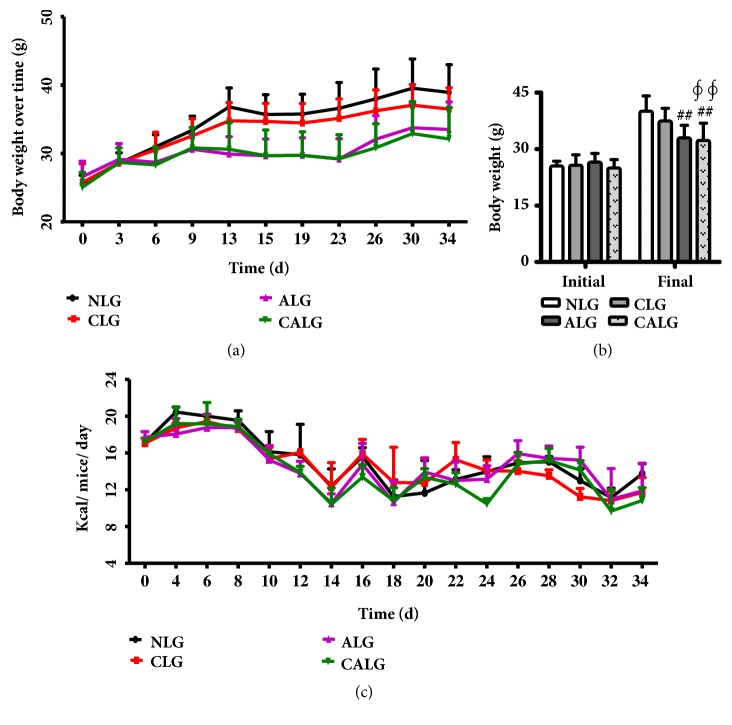
*Body weight change and caloric consumption during the 5-week experiment.* (a) Body weight change over time. (b) The initial and final body weight. (c) Caloric consumption during the experiment. Values were expressed as the mean ± SD (n=12). ^##^ *P* < 0.01 versus NLG; ^*∮∮*^ *P* < 0.01 versus CLG.

The four dietary groups' mice had similar baseline weight (25.72±2.13g). By the end of experiment, there was no difference of mice weight between the NLG and CLG. In contrast, mice gained less weight in ALG and CALG compared with the NLG (*P *< 0.01) (Figures 2(a) and 2(b)). It indicated that the addition of cholesterol caused less weight gain in the presence of increased dietary 4% alcohol, with the same calories intake among the four dietary groups mice pair-fed (Figure 2(c)).

### 3.2. Alcohol with Cholesterol Diet Causes Increasing Serum Levels of Liver Enzymes and Fasting Lipids

**Figure 3:**
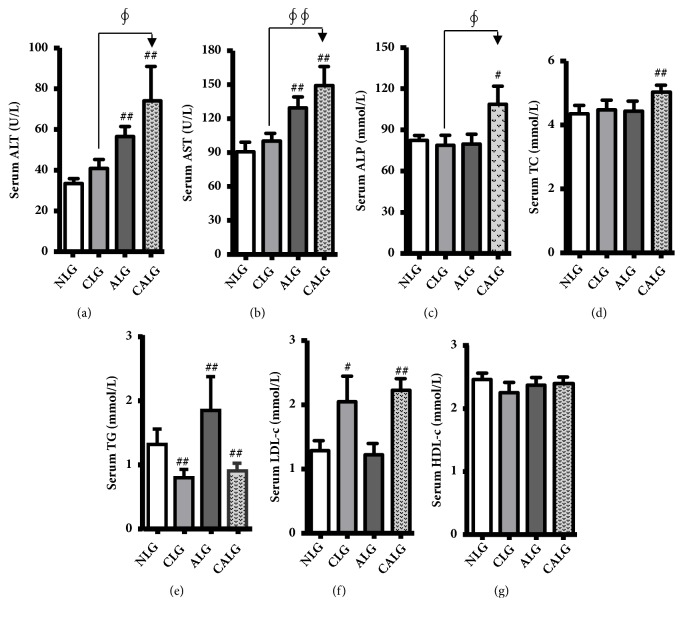
*Mice fed with 4% alcohol and 0.5% cholesterol LD increasing serum levels of liver enzymes and fasting lipids for feeding 5 weeks.* (a, b, and c) Liver damage reflected by levels of serum ALT, AST, and ALP. (d, e, f, and g) Serum lipids of TC, TG, HDL-c, and LDL-c were detected. Values were expressed as the mean ± SD (n=12). ^#^ *P* < 0.05; ^##^ *P* < 0.01 versus NLG; ^*∮*^ *P* < 0.05; ^*∮∮*^ *P* < 0.01 versus CLG.

Serum ALT, AST, and ALP level were markers of hepatocyte necrosis. In our experiments, serum ALT was normal in the NLG (33.45±7.75 U/L), very mildly elevated in the CLG (40.83±15.30 U/L), significantly increased in the ALG and CALG, with almost 2-fold elevated in the CALG (*P *< 0.01) and serum ALT in the CALG significantly increased compared with the CLG (*P *< 0.05) (Figure 3(a)). Meanwhile, serum AST changed, as well as compared with the NLG or CLG, was the same tendency like ALT in all experimental groups after feeding 5 weeks (*P *< 0.01) (Figure 3(b)). Serum ALP also significantly increased in the CALG compared with the NLG or CLG (*P *< 0.05) (Figure 3(c)).

What is more, serum TG was significantly elevated in the ALG, but there were the opposite results in the CLG and CALG compared with the NLG (*P *< 0.01) (Figure 3(e)). Serum TC and LDL-c were all significantly increased in the CALG and only serum LDL-c was significantly increased in the CLG compared with the NLG (*P *< 0.01, 0.05) (Figures 3(d) and 3(f)). There was no significant change in serum HDL-c in all groups (Figure 3(g)). It indicated that the combination of alcohol and cholesterol diet induced more severe liver injury and disorder of lipid metabolism in mice.

### 3.3. Alcohol with Cholesterol Diet Causes Greater Hepatic Lipid Accumulation and Increase Liver Tissue Histological Inflammation

**Figure 4:**
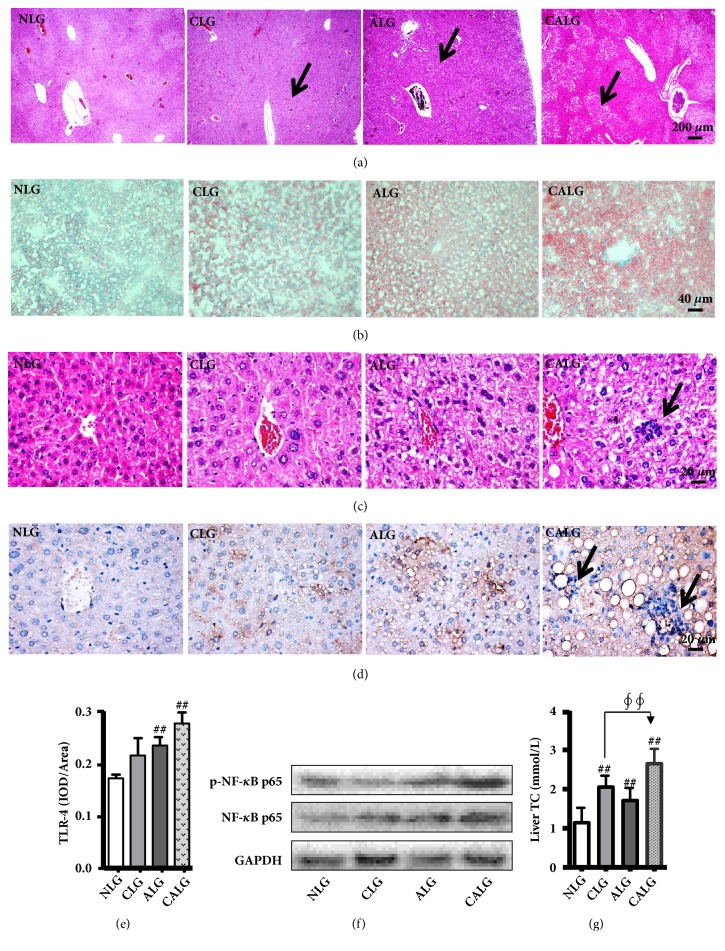
*Mice fed 4% alcohol and 0.5% cholesterol LD causes greater hepatic lipid accumulation and increases liver tissue histological inflammation for feeding 5 weeks.* (a and c) Liver damage directly reflected by H&E (x 40 and x 400). (b) Oil Red O staining shows the excessive cytoplasmic lipid accumulation (x 200). (d) Immunohistochemistry reflected the expression of TLR4 (x 400). (e) The data of TLR4 expression was semiquantitatively analysed as integrated option density (IOD) in positive area of the microphotograph. (f) Western blot reflected the expression of NF-*κ*B and p-NF-*κ*B. (g) Hepatic lipid accumulation directly reflected by the level of TC in liver. (a–f) Alcohol induced more severe liver injury and steatosis with cholesterol ingestion. Values were expressed as the mean ± SD (n=3), ^#^ *P* < 0.05; ^##^ *P* < 0.01 versus NLG. (g) Values were expressed as the mean ± SD (n=12), ^#^ *P* < 0.05; ^##^ *P* < 0.01 versus NLG; ^*∮*^ *P* < 0.05; ^*∮∮*^ *P* < 0.01 versus CLG.

In order to examine whether alcohol ingestion developed more severe liver injury and steatosis at the present of cholesterol, we further observed pathological changes in liver tissues of mice stained with the H&E and Oil red O. The results showed that cholesterol and alcohol diet could induce severe steatosis, respectively (Figures 4(a)–4(c)). In contrast, it was more severe liver injury, steatosis, and the level of TC in liver in the CALG (Figures 4(a)–4(c) and 4(g)). Additionally, there was liver histological inflammation observed in the CALG after experiments for 5 weeks (Figures 4(c) and 4(d)). Further research found that TLR4 and NF-*κ*B p65 were also high expression in the CALG compared with the NLG (Figures 4(d)–4(f)). These data indicated that alcohol induced more severe liver injury and steatosis with cholesterol ingestion in mice.

### 3.4. Dietary Alcohol Exacerbates Hepatic Lipid Loading by Increasing Cholesterol Intake and Syntheses and Reducing Cholesterol Conversion

To understand whether alcohol ingestion induces more severe liver damage by influence cholesterol metabolism, many proteins, correlated to cholesterol intake, syntheses and conversion, were measured.

**Figure 5:**
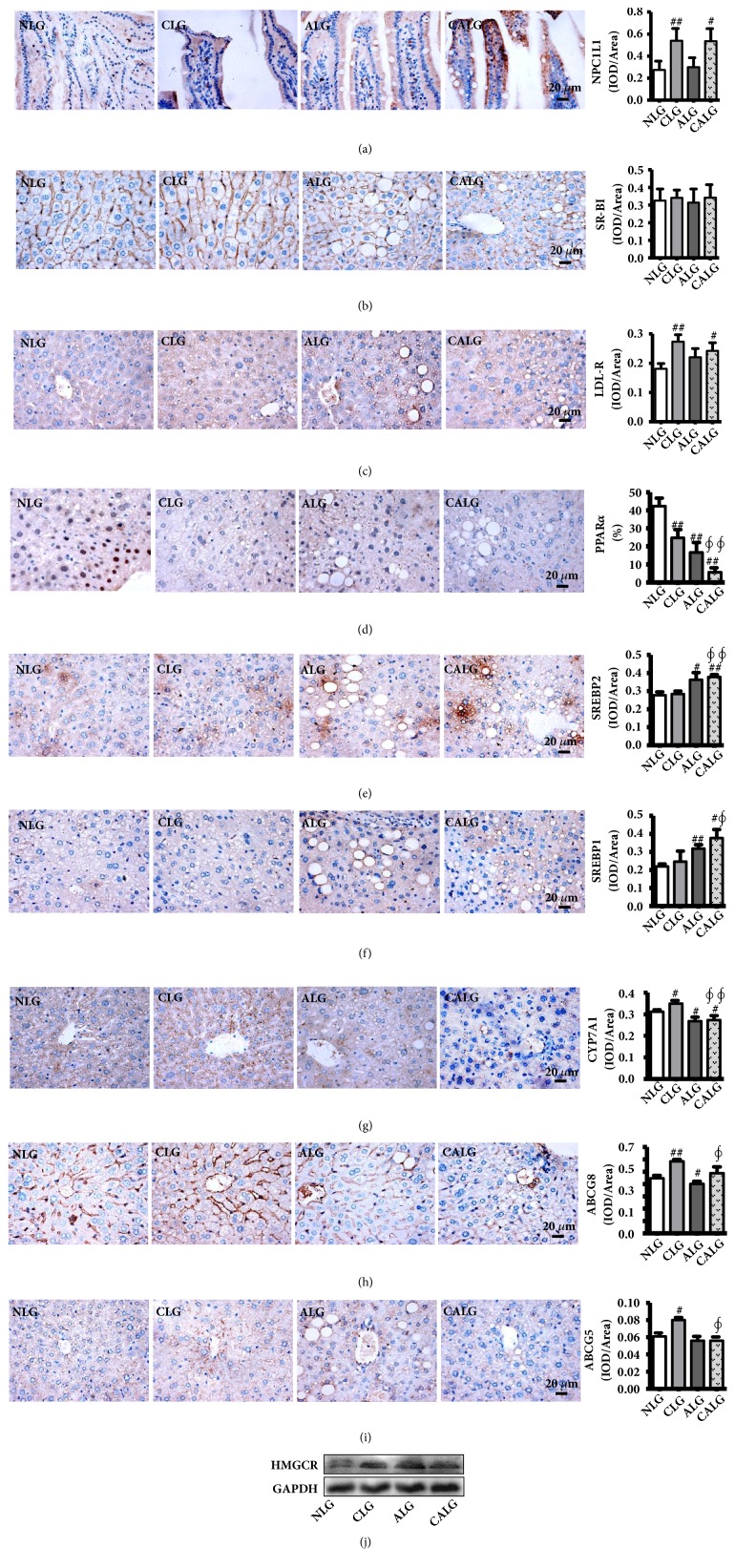
*Mice fed 4% alcohol and 0.5% cholesterol LD impacted cholesterol metabolism in liver for feeding 5 weeks.* (a–i) Immunohistochemistry reflected the expression of LDLR, PPAR*α*, SREBP2, SREBP1, CYP7A1, ABCG5, and ABCG8 in liver and NPC1L1 in small intestine (x 400). (j) Western blot reflected the expression of HMGCR in liver. Values were expressed as the mean ± SD (n=3). ^#^ *P *< 0.05; ^##^ *P *< 0.01 versus NLG; ^*∮*^ *P *< 0.05; ^*∮∮*^ *P *< 0.01 versus CLG.

Cholesterol was firstly absorbed into the body's metabolism in the small intestine through NPC1L1 and then may enter the liver metabolism in the form of LDL-c and HDL-c through LDLR and SR-BI, respectively. The IHC results show that the expression NPC1L1 in the small intestine and LDLR in the liver significantly increased in the CLG and CALG (*P* < 0.05, 0.01) and there was no significant change in SR-BI in the liver between all groups (Figures 5(a)–5(c)).

HMGCR is the rate-limiting enzyme of cholesterol synthesis in liver, which resulted in the regulation of PPAR*α* and SREBP1/2. Compared with NLG, the hepatic IHC staining showed that the expression of SREBP-2 and SREBP-1 was significantly upregulated in ALG and CALG (*P* < 0.05, 0.01) (Figures 5(e) and 5(f)). And the expression of PPAR*α* was markedly downregulated in CLG, ALG, and CALG (*P* < 0.01) (Figure 5(d)). Meanwhile, the result of liver western blot showed that the protein expression of HMGCR was apparently increased ALG and CALG (Figure 5(j)). These results demonstrated that cholesterol and alcohol ingestion increased liver cholesterol synthesis in mice.

In the liver, ABCG5/ABCG8 drives the hepatic cholesterol into the bile promoting cholesterol excretion, and CYP7A1 is the rate-limiting enzyme in the synthesis of bile acid from cholesterol. Compared with the NLG, the expression of CYP7A1 in the liver was significantly increased in the CLG (*P* < 0.01), but was significantly decreased in the ALG and CALG (*P* < 0.01) (Figure 5(g)). And the expressions of ABCG5/ABCG8 were significantly upregulated in the CLG. In contrast, there was also an opposite result of ABCG5/ABCG8 between the ALG and CALG (Figures 5(h) and 5(i)). The present results illustrated that alcohol ingestion suppressed the liver cholesterol excretion in mice.

### 3.5. Inhibition Cholesterol into the Body's Metabolism in the Small Intestine Significantly Improves the Fatty Liver Disease the FLD Rats

**Figure 6:**
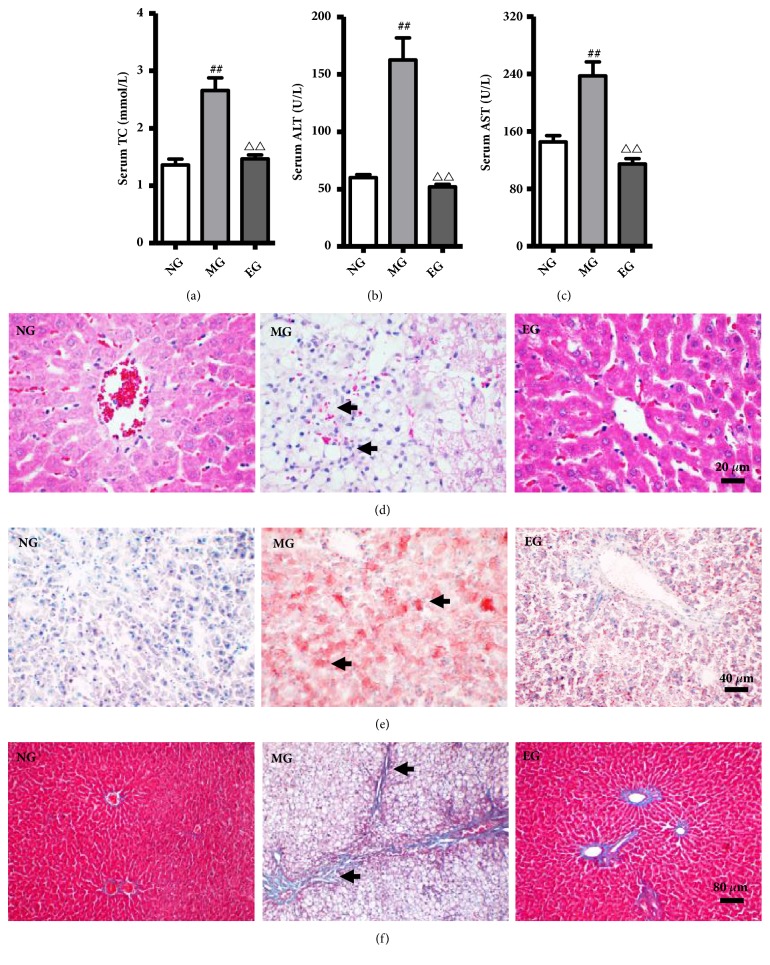
*Ezetimibe alleviated liver lesion in the high fat-cholesterol-sucrose and alcohol- (ACHFCSD-) induced rat model.* (a) Serum level of TC. (b and c) Serum level of ALT and AST. (d) H&E staining of liver (x 400). (e) Oil Red O staining of liver (x 200). (f) Masson staining of liver (x 100). Values were expressed as the mean ± SD (n=10). ^##^ *P *< 0.01 versus Normal group; ^△△^ *P *< 0.01 versus Model group.

Ezetimibe, a selective inhibitor of NPC1L1 in small intestinal mucosa, can inhibit exogenous cholesterol absorption. To understand whether alcohol ingestion induces more severe liver damage by influence cholesterol metabolism, we observed the effect of Ezetimibe in SD rats fed with high fat-cholesterol-sucrose and alcohol. The levels of serum TC, ALT, and AST in the MG were significantly elevated compared to the normal control rats (*P *< 0.01) (Figures 6(a)–6(c)). Subsequently, the liver sections were stained with H&E, Oil Red O, and Masson, and the results revealed that ballooning degeneration steatosis, inflammatory cell infiltration around the pericentral zone, and lipid accumulation were obviously increased in the MG (Figures 6(d)–6(f)). In contrast, Ezetimibe supplementation could significantly reverse these lesions induced by the ACHFCSD. These data indicated that inhibition cholesterol into the body's metabolism in the small intestine significantly improves the fatty liver disease induced by the ACHFCSD.

## 4. Discussion

A high alcohol or cholesterol diet is well-established risk factors of FLD in humans, which is a major cause of morbidity and mortality worldwide. The spectrum of FLD comprises steatosis, steatohepatitis, progressive fibrosis, and hepatocellular carcinoma, and it is influenced by numerous factors including lifestyle and habits such as alcohol abuse, high-fat diet, and high-cholesterol diet. The intake of more than 30 g of absolute alcohol per day could increase the risk of alcoholic liver disease. Dietary cholesterol, possibly in the form of modified plasma lipoproteins, is an important risk factor for the progression to hepatic inflammation in diet-induced liver disease. However, whether and how alcohol consumption simultaneously with cholesterol ingestion induces more severe FLD remain unclear.

In our study, there was no significant change in the cholesterol diet group and alcohol diet group after 3 weeks, while TC significantly increased in the cholesterol and alcohol diet group. Similar result was also observed up until 5 weeks modeling. Moreover, while the low-density lipoprotein-cholesterol (LDL-c) concentration did not change in the alcohol diet group, it significantly increased in the cholesterol diet group, and increased even more in the cholesterol and alcohol diet group. As mice lack the enzyme cholesteryl ester transfer protein (CETP) [15], 70% of the cholesterol in serum is stored in the form of LDL-c; with the intake of cholesterol, alcohol could lead to an increase in the level of TC and LDL-c, which would indicate that alcohol can influence cholesterol metabolism in mice. Alcohol affecting lipid metabolism may be mainly by inhibiting the expression of PPAR*α* and AMP-activated protein kinase (AMPK), which also induce severe FLD [16–18]. Alcohol can reduce the binding of PPAR*α* with RXR DNA, reducing the expression of some PPAR*α* regulatory genes, such as downregulation of the expression of MTP leading to the oxidation and transport of fatty acids [16, 17, 19]. Alcohol can also inhibit AMPK activity [16, 19], thus upregulating the downstream regulatory element SREBPs, and then activate the genes expression promoting fatty acid and cholesterol synthesis [18].

**Figure 7:**
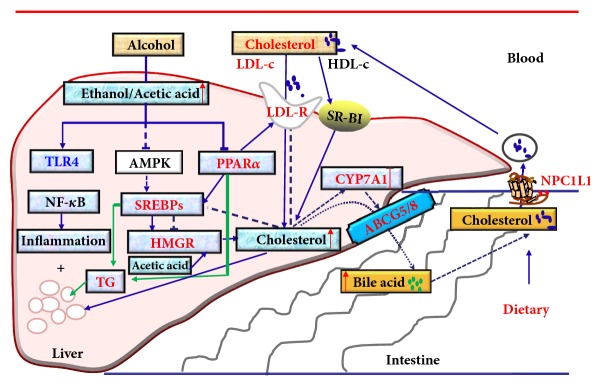
Mechanism of alcohol affecting cholesterol metabolism and aggravating liver damage.

Significant increase in the serum levels of the marker ALT, AST, and ALP is conventional indicator of liver injury [20]. Previous studies have shown that mice and rats exhibit higher levels of serum ALT, AST, or ALP upon alcohol consumption [21, 22]. However, it was unclear whether a normal diet containing more cholesterol and alcohol leads to higher levels of serum ALT, AST, and ALP in these animals. The results of our study demonstrated that 4% alcohol and 0.5% cholesterol ingestion resulted in even higher level of serum ALT, AST, and ALP in mice. The more severe liver damage in the alcohol and cholesterol diet group was also manifested as changes of liver morphology and pathology. Alcohol may damage the liver not only through oxidative stress, mitochondrial injury, and acetaldehyde toxicity [23], but also through lipid deposition and lipid oxidative damage [16–18] like cholesterol. Those findings indicated that alcohol together with cholesterol intake had induced more severe liver injury. To investigate how alcohol influenced cholesterol metabolism so to induce more severe liver damage, we analyzed the expression of proteins associated with the three main mechanisms that affect cholesterol metabolism, including NPC1L1, SR-BI, PPAR*α*, LDLR, SREBP1/2, CYP7A1, and ABCG5/8 (Figure 7).

SREBPs (including SREBP1*α*, SREBP1c, and SREBP2) are transcription factor that regulate the synthesis of fatty acids, triglycerides, and cholesterol and specifically bind to the cholesterol regulatory element 1 (SRE-1). SREBP2 mainly promotes the synthesis and uptake of cholesterol [24]. Chronic alcohol intake can promote SREBP1c contents [25] and has no significant effect on SREBP2 [26]. Research has also shown that alcohol activates SREBP1 by inhibiting AMPK and influences the synthesis of cholesterol [18]. When the level of cholesterol increases, SREBPs will not be activated and cannot be bind to target gene promoter of the cholesterol regulatory element (SRE), resulting in transcriptional inhibition of LDLR [27] or HMGR [28], which is the rate-limiting enzyme for cholesterol synthesis. Additionally, alcohol intake can inhibit PPAR*α* [25] and activation of PPAR*α* reduces the synthesis and concentration of cholesterol by decreasing the level of SREBP2, which means maybe to say that inhibition of PPAR*α* increases the level of SREBP2 [29]. In our study, the expression of SREBP1, SREBP2, and HMGR was evaluated and PPAR*α* was more notably decreased in the alcohol and cholesterol diet group, which indicated that alcohol may promote cholesterol synthesis and increase the accumulation of cholesterol in the liver by affecting the PPAR*α*-SREBPs-HMGR pathway.

Blood cholesterol may deliver to the liver by LDL through the LDLR of hepatocytes. Most of cholesterol absorbed in food is transported into liver in the form of LDL-c. When the LDLR expression is increased, more cholesterol is transported into the liver [30], thus causing more severe hepatic cholesterol accumulation (Figure 4(g)), further aggravating the injury. Unlike some previous reports [31, 32], our study found that the cholesterol diet alone or combined with the alcohol diet all could increase the expression of the liver LDLR in mice. The different results may be related to the molding species, diet, and time in those previous reports, which were involve with guinea pigs and C57 mice, high-fat-cholesterol diet, and molding for 12 or 6 weeks [31, 32]. And there was also report that the expression of LDLR increase in liver of high-fat diet-induce mice [33]. The metabolic pathway of LDLR can be adjusted by LXR-Idol-LDLR axis [30], LDLR-PCSK9 axis [34], and PPARa-LDLR axis [35]. As plasma LDL particles could dose-dependent blunt PCSK9-mediated LDLR degradation by binding to PCSK9 [34], and activation of PPARa in mice induced expression of the hepatic LDLR [35]. In our study, the cholesterol diet alone or combined with the alcohol diet all could increase serum LDL-c and decrease the liver PPARa, which may the main cause of the high expression of the liver LDLR in those mice.

CYP7A1 is an important cytochrome P450 monooxygenase that catalyzes the reaction which transforms cholesterol into bile acids. The cholesterol diet feedback increased the expression of CYP7A1 [36], thus reducing the cholesterol levels in the liver. Alcohol consumption has been reported to reduce the expression of the CYP7A1 and alcohol-induced hepatic inflammation and injury were ameliorated in CYP7A1 transgenic mice [37], but contrasting results have also been reported in previous studies [38–40]. ABCG5 and ABCG8 are important proteins in the excretion of bile acid produced by the liver. In previous studies, the cholesterol diet increased the expression of ABCG5/8 in the liver promoting biliary cholesterol secretion [41], to reduce excessive cholesterol accumulation in the liver. Alcohol feeding reduced ABCG5 and ABCG8 gene expression in wild-type mice [37]. However, there has been little research on the impact of cholesterol combined with alcohol on the expression of CYP7A1 and ABCG5/8. In our experiment, the cholesterol diet increased the expression of ABCG5/8 and CYP7A1 and the alcohol diet repressed their expression, which is consistent with the previous reports [36, 37, 40, 41]. In comparison, the cholesterol diet combined with the alcohol diet had a similar effect on the expression of ABCG5/8 and CYP7A1 as the alcohol diet in mice. It may be that the inhibitory effect of alcohol on the expression of ABCG5/8 and CYP7A1 counteracts the increased effect of cholesterol on them. Accordingly, by reducing the cholesterol transformation into bile acid and inhibiting the excretion of cholesterol, the alcohol diet combined with the cholesterol diet aggravated liver cholesterol accumulation.

The metabolic disorder of cholesterol often causes accumulation of other lipids in the liver, especially triglyceride (TG) metabolism, thus further aggravating the lipid accumulation in liver. The results of the Oil Red O staining experiment proved our hypothesis that the accumulation of lipid droplets in the alcohol and cholesterol diet group was more severe. It has been found that ethanol inhibits the expression of AMPK and PPAR*α*, as well as the oxidation of fatty acids and increases the synthesis of fatty acids. The increase of fatty acid synthesis and the decrease of oxidation lead to larger amounts of free fatty acids entering the TG anabolic pathway, which in turn significantly increases the synthesis of TG [18, 25]. The results of our experiment showed that the serum TG were reduced by the cholesterol diet alone or combined with alcohol in mice, but it was significantly increased after alcohol intake alone. The changes in serum TG levels induced by chronic alcohol were different, which may be related to the animal species, modeling time, and modeling methods [25, 42]. It has also been reported that the cholesterol diet can cause lipid metabolism disorder in rats and mice, with decreased serum TG and increased serum TC and as well as increased the concentrations of hepatic TC and TG [43]. The results of the H&E pathological tissue staining also showed that the hepatic steatosis was serious, and even the inflammatory lesions appeared in animals of the cholesterol and alcohol diet group.

Cholesterol is initially absorbed into the body's metabolism in the small intestine through NPC1L1 [44]. Our results showed that the cholesterol-rich diet alone or combined with the alcohol consumption also significantly increased the expression NPC1L1 in the small intestine. Moreover, we selected the Ezetimibe, a selective inhibitor of NPC1L1 in small intestinal mucosa, and verified that inhibition of intestinal cholesterol absorption, but not the absorption of fat, sucrose, and alcohol into the body's metabolism, could significantly improve FLD in rats fed with high fat-cholesterol-sucrose and alcohol. Those data indicated that cholesterol metabolism occupies an important position in cholesterol and alcohol-induced FLD. The results indicated that cholesterol metabolism plays an important role in cholesterol and alcohol-induced FLD.

Additional mechanisms whereby alcohol might promote hepatic inflammation are also possible. The potential mechanisms include that alcohol destroys the natural barrier of the intestines and increases permeability of the intestinal mucosa. As a result, LPS gets into enterohepatic circulation, which leads to activation of the TLR4/NF-*κ*B pathway in the liver Kupffer cells, and induces the release of large inflammatory factors that ultimately result in liver damage. In rats, dietary alcohol has been suggested to cause Kupffer cells activation, leading to inflammatory infiltrate [45]. In addition, it was demonstrated that in our experiments the setting of alcohol and cholesterol diet causes more inflammatory infiltrate than alcohol or cholesterol diet alone.

In conclusion, the cholesterol diet combined with the alcohol diet-induced FLD may synthesize the risk factors of alcoholic and nonalcoholic fatty liver disease and inhibit some beneficial feedback regulation mechanisms to accelerate and aggravate the occurrence and development of FLD. The present study demonstrated that alcohol consumption, together with cholesterol ingestion, induces more severe FLD by influencing cholesterol metabolism, which might be mainly related to influence of cholesterol absorption (LDLR↑ and NPC1L1↑), synthesis (PPAR*α*↓, SREBP1/2↑, and HMGCR↑), and excretion (CYP7A1↓ and ABCG5/8↓) in liver or small intestine. Our findings can indicate that the combination of alcohol consumption and high-cholesterol diet for a long time is very bad, as it will accelerate the development of FLD. The investigation of the mechanisms has certain significance for the prevention and treatment of FLD. In the future research, it needs use of more experimental methods to validate the significantly changed indicators, and more animal models and different modeling time should be considered to illustrate the differences of some indicators, or with the clinical samples.

## Figures and Tables

**Table 1:** Caloric profile of the Lieber-DeCarli diet (k cal per liter).

*Ingredients*	*NLG*	*CLG*	*ALG*	*CALG*
Protein	180	180	180	180
Fat	350	350	350	350
Carbohydrate	470	470	182	182
Alcohol	0	0	288	288
Total	1000	1000	1000	1000

## Data Availability

The data used to support the findings of this study are available from the corresponding author upon request.

## Supplementary Materials

Supplementary Materials Figure S1: serum TC level of mice fed with 4% alcohol and 0.5% cholesterol LD after feeding 3 weeks and liver TC content after feeding 5 weeks. Serum TC was detected by an automatic biochemical analyzer and liver TC content was measured by total cholesterol assay kit (purchased from NanJing JianCheng Bioengineering Institute) according to instructions.
