# Supplementary material for: Alcohol Induces More Severe Fatty Liver Disease by Influencing Cholesterol Metabolism
Source: Evid Based Complement Alternat Med. 2019 Feb 12;2019:7095684. doi: 10.1155/2019/7095684 (PMC6390266; doi:10.1155/2019/7095684)
Supplement: Supplementary Materials — Figure S1: serum TC level of mice fed with 4% alcohol and 0.5% cholesterol LD after feeding 3 weeks and liver TC content after feeding 5 weeks. Serum TC was detected by an automatic biochemical analyzer and liver TC content was measured by total cholesterol assay kit (purchased from NanJing JianCheng Bioengineering Institute) according to instructions. [file 7095684.f1.pdf]

A

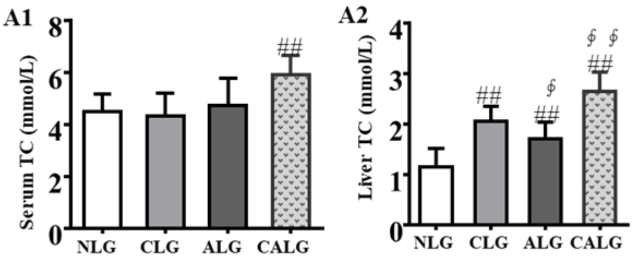

Fig.S1. Mice fed with 4% alcohol & 0.5% cholesterol LD increased serum TC after feeding 3 weeks (**A1**) and liver levels of TC (**A2**) after feeding 5 weeks. Values were expressed as the mean ± SD (n=6). $^{\#}p<0.05$, $^{\#\#}p<0.01$ vs NLG; $^{\$}p<0.05$, $^{\$\$}p<0.01$ vs CLG.
